# Dysfunctional Innate Immune Responses and Severe Dengue

**DOI:** 10.3389/fcimb.2020.590004

**Published:** 2020-10-23

**Authors:** Gathsaurie Neelika Malavige, Chandima Jeewandara, Graham S. Ogg

**Affiliations:** ^1^Centre for Dengue Research, Faculty of Medical Sciences, University of Sri Jayewardenepura, Nugegoda, Sri Lanka; ^2^MRC Human Immunology Unit, MRC Weatherall Institute of Molecular Medicine, University of Oxford, Oxford, United Kingdom

**Keywords:** dengue, innate immunity, monocytes, mast cells, antibody dependent enhancement, endotoxin, T cells, B cells

## Abstract

Although infection with the dengue virus (DENV) causes severe dengue, it causes a mild self-limiting illness in the majority of individuals. There is emerging evidence that an aberrant immune response in the initial stages of infection lead to severe disease. Many inflammatory cytokines, chemokines, and lipid mediators are significantly higher in patients with severe dengue compared to those who develop mild infection, during febrile phase of illness. Monocytes, mast cells, and many other cells of the immune system, when infected with the DENV, especially in the presence of poorly neutralizing antibodies, leads to production of pro-inflammatory cytokines and inhibition of interferon signaling pathways. In addition, production of immunosuppressive cytokines such as IL-10 further leads to inhibition of cellular antiviral responses. This dysregulated and aberrant immune response leads to reduced clearance of the virus, and severe dengue by inducing a vascular leak and excessive inflammation due to high levels of inflammatory cytokines. Individuals with comorbid illnesses could be prone to more severe dengue due to low grade endotoxemia, gut microbial dysbiosis and an altered phenotype of innate immune cells. The immunosuppressive and inflammatory lipid mediators and altered phenotype of monocytes are likely to further act on T cells and B cells leading to an impaired adaptive immune response to the virus. Therefore, in order to identify therapeutic targets for treatment of dengue, it would be important to further characterize these mechanisms in order for early intervention. In this review, we discuss the differences in the innate immune responses in those who progress to develop severe dengue, compared to those with milder disease in order to understand the mechanisms that lead to severe dengue.

## Introduction

Dengue infections represent one of the most important vector-borne diseases in the world, resulting in significant morbidity and mortality. It is estimated than 105 to 390 million individuals are infected with one of the four dengue viruses (DENV) annually leading to 51 to 96 million apparent dengue infections (Bhatt et al., 2013; Cattarino et al., 2020). Although the infection is asymptomatic or mild in most infected individuals, it can lead to severe forms of disease such as dengue hemorrhagic fever (DHF) and death in some individuals (Cattarino et al., 2020). DHF is shown to occur in ~23.2% of symptomatic individuals, and if untreated, is reported to have case fatality rates (CFRs) of ~20% (Guo et al., 2017). Although CFRs are <1% in most countries due to intense monitoring of patients who develop dengue and meticulous fluid management, CFRs are reported to be around 2.6% in some countries (Guo et al., 2017; Murhekar et al., 2019).

Initial infection with a particular DENV serotype is known as a primary infection, while subsequent infection with any other serotype is known as a secondary dengue infection (WHO, 2011). Secondary dengue infection is thought to be one of the most important risk factors for development of severe disease, in part due to antibody dependent enhancement (ADE) in which poorly neutralizing, highly cross-reactive antibodies enhance infection in FcγR-expressing cells (Chareonsirisuthigul et al., 2007; Chau et al., 2008; Guzman et al., 2013; Syenina et al., 2015; Katzelnick et al., 2017). However, not all secondary dengue infections result in symptomatic infection or DHF and only 15% of secondary dengue infections progress to DHF (Wang et al., 2017). Subclinical dengue infection occurs in an equal proportion of those experiencing a primary or secondary dengue infections (Grange et al., 2014). Therefore, many host factors apart from the presence of poorly neutralizing cross-reactive antibodies could lead to protection or pathogenesis.

In those who develop a symptomatic illness, there can be sudden onset of fever along with headaches, myalgia and arthralgia. This initial febrile phase (early illness) typically lasts for 3 to 6 days and the patient recovers in most instances. However, ~25% of individuals progress to develop complications around day 3 to 6 of illness, which is characterized by fluid leakage leading to pleural effusions, ascites, shock, organ dysfunction and if untreated, can lead to death (WHO, 2011; Malavige and Ogg, 2017). There is emerging evidence that an aberrant immune response in the initial stage of infection, results in endothelial dysfunction and cytokine storms leading to vascular leakage and thus DHF. In this review, we discuss the differences in the innate immune responses in those who progress to develop DHF compared to those with milder disease in order to understand the mechanisms that lead to severe dengue.

## Cytokine and Chemokines Signatures in Early Illness Leading to Severe Illness

Many different chemokines and cytokines have been shown to be elevated in severe dengue such as IFN-γ, GM-CSF, IL-10, MIP-1β, IL-1β, IL-8 TNFα, IP-10, MCP-1, and IL-18 (Bozza et al., 2008; Malavige et al., 2013a; Fernando et al., 2016; Kamaladasa et al., 2016; van Wilgenburg et al., 2016; Patro et al., 2019). Certain cytokines such as TNFα and IL-1β have shown to directly cause vascular leak (Hottz et al., 2014; Kamaladasa et al., 2016), while both these cytokines along with IL-18, IP-10, IL-8, and MIP-1β are potent inflammatory cytokines produced by many immune cells (Tang et al., 2020). IL-10 on the other hand is a potent immunosuppressive cytokine, which has been associated with severe dengue (Malavige et al., 2013a,b). Due to the presence of high levels of these cytokines during the critical phase and due to the presence of cross reactive DENV-specific T cells during acute dengue, T cells were implicated as the main source of these cytokines and the drivers of the cytokine storm (Mongkolsapaya et al., 2006; Appanna et al., 2007; Dong et al., 2007). However, more recent data have shown that innate immune cells such as monocytes are one of the main sources of such cytokines and chemokines, while DENV-specific T cells are likely to be protective (Weiskopf et al., 2013, 2015; Singla et al., 2016; Wijeratne et al., 2018). Those who proceeded to develop severe disease had higher levels of many types of inflammatory markers such a C-reactive protein, increased inflammatory lipid mediators and cytokines in very early illness (initial period of the febrile phase), before they proceeded to develop severe disease or vascular leak (Fernando et al., 2016; Jeewandara et al., 2017; Vuong et al., 2020). For instance, CRP levels of >34 mg/L within the first 3 days of illness (before any patients had developed severe disease) was associated with an increased risk of progression to severe illness (Vuong et al., 2020). Similar findings were observed with an inflammatory lipid enzyme mediator sPLA2, where the enzyme activity was highest during early illness in those progressed to develop DHF (Jeewandara et al., 2017).

Although cytokines and chemokines have been extensively studied in in dengue, only a few have studied their changes during different clinical phases of dengue. Due to the dynamic changes in the clinical and laboratory features along with cytokines and chemokines during the febrile phase (before the onset of vascular leak), is likely to be different than those in the critical phase. A study carried out in a relatively small sample of patients showed that IP-10, MCP-1, and MIP-1β were significantly higher in those who progressed to develop plasma leakage, again during the febrile phase compared to the critical phase (Rathakrishnan et al., 2012). A more recent study, done in a large cohort of patients showed that while many cytokines are elevated in patients with dengue during the febrile phase, it was IL-10 that most significantly associated with subsequent development of DHF (Dayarathna et al., 2020, under review). Therefore, collectively these data suggest that the events that trigger the release of these inflammatory mediators occur very early in illness (initial period of the febrile phase), potentially due to the differences in the innate immune responses to the virus.

Several proteins of the DENV have been shown to inhibit IFN signaling pathways, while NS1, which is a secretory protein has shown to directly cause disease pathogenesis (Castillo Ramirez and Urcuqui-Inchima, 2015; Modhiran et al., 2015; Adikari et al., 2016; Puerta-Guardo et al., 2016; Kao et al., 2018). NS1 Ag, which exists in a hexametric form, has been shown to trigger cytokine release from PBMCs through binding to TLR4 and also to contribute directly to vascular leak by disruption of the endothelial glycocalyx (Modhiran et al., 2015; Glasner et al., 2017). NS1 is a major target of antibodies that develop during natural DENV infections (Dejnirattisai et al., 2010) and mice immunized with NS1 or given polyclonal sera of mice immunized with NS1 were shown to have significantly less vascular leak, when challenged with the DENV (Beatty et al., 2015). However some studies have shown that NS1-NS1 antibody complexes contribute to vascular leak by activation of complement and by causing endothelial apoptosis (Avirutnan et al., 2006). Studies in acute dengue have shown that NS1 antibody levels rise in those with severe dengue compared to those with milder forms of illness, during acute illness and that those with milder dengue appeared to recognize different epitopes than those with severe illness (Jayathilaka et al., 2018). The lower than expected efficacy of some dengue vaccines, have been attributed to lack of generation of NS1 antibodies (Halstead, 2018,Nascimento et al., 2018).

The non-structural proteins NS2A and NS3 have been shown to inhibit type I interferon production by degrading the stimulator of interferon genes (STING) and IRF3, which in turn impairs RIG-I and TLR-3 signaling pathways (Castillo Ramirez and Urcuqui-Inchima, 2015). Both NS3, NS2A along with NS4A, NS4B, and NS5 also further inhibit interferon signaling by preventing STAT-1 phosphorylation (Castillo Ramirez and Urcuqui-Inchima, 2015; Kao et al., 2018). DENV strains which produce higher levels of subgenomic RNA and therefore are more capable of inhibiting TRIM-25 activation of RIG-I, have shown to have higher transmission rates and able to cause epidemics (Manokaran et al., 2015). Therefore, inhibition of IFN production appears to be an important mechanism of immune evasion by the DENV, which is associated with increased clinical disease severity and epidemiological fitness. However, inhibition of IFN signaling by the DENV does not explain the occurrence of severe disease in some individuals, and mild/asymptomatic infection in others, who are infected with the same strain of the DENV. It is possible that those who develop more severe illness have higher viral replication within the host cells due to increased viral entry into such cells due to ADE and/or other genetic or immunological factors which influence the type I IFN response, and therefore, more inhibition of IFN signaling pathways.

## Monocyte/Macrophage Responses in Severe Dengue

Although the DENV is known to infect many different types of cells, it was shown that monocytes are the cells most commonly infected by the virus (Zanini et al., 2018). Single cell transcriptomics in PBMCs of patients during early illness, before they progressed to severe disease, showed that cells of those who subsequently had severe disease had a unique transcriptomic signature (Zanini et al., 2018). Of the gene signatures associated with progression to severe disease, *IFITI* and *CD163* expression in CD14^+^CD16^+^ monocytes and *MX2* in B lymphocytes had the highest predictive value, highlighting the important role of monocytes in disease pathogenesis (Zanini et al., 2018). Soluble CD163 which is a marker of macrophage activation syndrome has been previously shown to differentiate those who have severe dengue from DF (Ab-Rahman et al., 2016). Therefore, macrophage activation associated with various cytokines such as TNFα, IL-6, and IL-10 or by oxidative stress appears to be a feature of DHF.

**Figure 1:**
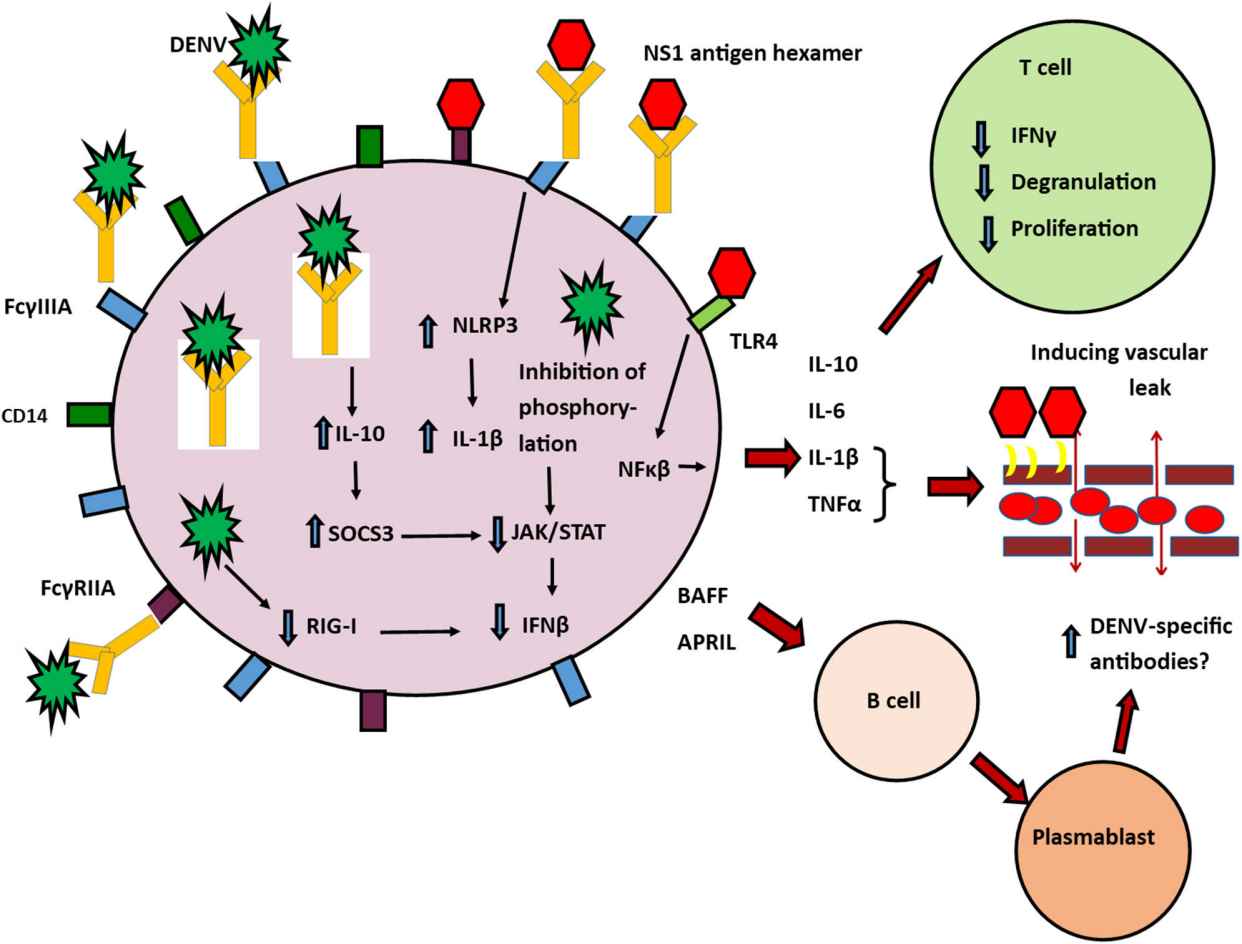
Possible role of monocytes in pathogenesis of severe dengue. Classical and non-classical monocytes have many types of activating FcγRs, that facilitate antibody dependent enhancement (ADE) such as FcγRI, FcγRIIA, and FcγRIIA. FcγRI and FcγRIIA downstream signaling inhibits many antiviral responses by the cell, including inhibition of RIG-I/MDA5 induced type I IFN, TNFα, and IL-12 production. Non-classical (CD14^+^CD16^++^ monocytes) are more susceptible to infection through the DENV through ADE by binding of immune complexes to their FcγRIIIA (CD16a) receptors. This leads to induction of IL-10 and reduction of IFNβ and activation of iNOS. High levels of IL-10 induce SOCS3 which further inhibits JAK/STAT signaling pathways and inhibition of IFN production. DENV NS1 induced production of inflammatory cytokines after engagement through TLR-4 and contributes to vascular leak by disruption of the endothelial glycocalyx. Other DENV structural proteins leads to degradation of IFN signaling molecules resulting in impaired RIG-I signaling. DENV non-structural proteins also inhibit STAT-1 phosphorylation and thereby also contributing to reduction of IFN production. NS1 antibody-antigen immune complexes can also bind through FcγRIIIA (CD16a) receptors and induce NLRP3. This altered antiviral defense mechanisms within the cell leads to increased production of inflammatory cytokines and IL-10. IL-1β and TNFα contribute to vascular leak. IL-10 suppresses T cell activation, degranulation and cytokine production. These non-classical monocytes also produce BAFF and APRIL which act on resting B cells and stimulate them to transform into plasma cells, which are possibly responsible for further production of DENV-specific antibodies. 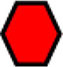 NS1 Hexamer, 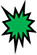 The DENV, 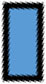 FcγRIIIA, 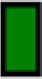 CD14, 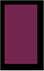 FcγRIIA, 
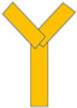
 antibodies, 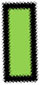 TLR4.

Apart from direct infection by the DENV, monocyte infection is further facilitated in acute dengue, by ADE (Chareonsirisuthigul et al., 2007; Syenina et al., 2015; Katzelnick et al., 2017). Internalization of antigen-antibody complexes is known to further suppress antiviral responses (Ubol et al., 2010; Tsai et al., 2014; Wang et al., 2017). *In vitro* infection of monocytes with the DENV, in the presence of sub-neutralizing concentrations of DENV-specific antibodies resulted in increased production of IL-10 and suppression of IFNβ and iNOS (Ubol et al., 2010). The high levels of IL-10 resulted in upregulation of negative regulators such as activation of suppressor of cytokine signaling system (SOCS), specifically SOCS-3 and inhibition of JAK-STAT pathways (Ubol et al., 2010) thereby suppressing antiviral defenses (Tsai et al., 2014) (Figure 1).

Certain FcγRs are more efficient at ADE than others. FcγRI, FcγRIIA, and FcγRIIIA are all shown to facilitate ADE while FcγRIIB is less efficient (Boonnak et al., 2008, 2013). FcγRIIA has shown to be more efficient than FcγRI in ADE in the presence of DENV-IgG immune complexes (Rodrigo et al., 2006). Downstream signaling pathways that are activated by engagement of the activating FcγRs (FcγRI and FcγRIIA), has shown to reduce production of TNFα, IL-12, and IFNβ through inhibition of interferon regulatory factor 1 and NFκB gene expression, while increasing the production of IL-10 (Ubol and Halstead, 2010). Engagement of immune complexes with the activating FcγRs in acute DENV infection results in activation of two suppression pathways, which leads to overall impairment of antiviral immune responses (Ubol and Halstead, 2010).

The FcγRIII receptor, which is another activating FcγR, which is important in ADE, has 2 isoforms, FcγRIIIA and FcγRIIIB (CD16a/CD16b). FcγRIIIA is known to bind to immune complexes more efficiently that other FcγRs (Ogonda et al., 2010). Increased expression of FcγRIIIA on non-classical monocytes (CD14^+^CD16^++^), has been linked to occurrence of severe anemia and other complications during *Plasmodium falciparum* infection (Ogonda et al., 2010). Higher levels of afucosylated Fc IgG1 isoforms, which have an enhanced capacity to engage with the activating FcRIIIA, was associated with DHF and shock in those with acute dengue suggesting antibody binding to FcγRIIIA is likely to be associated with severe disease (Wang et al., 2017). We recently showed that differences in antibodies to dengue NS1 antigen could also be associated with protection or severe disease (Jayathilaka et al., 2018). Indeed, in dengue mouse models, anti-NS1-Abs activated NLRP-3 by binding to FcγRIIIs resulting in severe disease (Lien et al., 2015). Therefore, it would be important to further investigate the role of different antibody subclass binding to different FcγRs and association with clinical disease severity.

Monocytes of healthy individuals who had varying severity of dengue in the past also appear to respond significantly different to infection with the DENV. We showed that monocytes of healthy individuals who had severe dengue in the past when infected with different serotypes of the DENV produced higher viral loads, higher levels of IL-10, IL-6, TNFα, and IL-1β and increased expression of RIG-I and NLRP3 when compared to monocytes of those with past non-severe dengue (Kamaladasa et al., 2019). These monocytes produced higher levels of IL-1β in the presence of autologous serum, in the presence of serum from those with past non-severe dengue and in the presence of seronegative serum suggesting that this aberrant responses by monocytes of those with past severe dengue could be an inherent characteristic (Kamaladasa et al., 2019). Although the reasons for this are not clear, it could be epigenetic reprograming in innate immune cells in such individuals due to exposure to different pathogens or other stimuli (Netea et al., 2016).

## Mast Cells in Dengue

Mast cells are shown to be permissive to infection by the DENV, which is enhanced in the presence of DENV-specific antibodies (St. John et al., 2013; Syenina et al., 2015). Many proteases and inflammatory mediators produced by mast cells such as chymases, tryptases, platelet activating factor (PAF) and vascular endothelial growth factor (VEGF) have been implicated in vascular leak and disease pathogenesis (Furuta et al., 2012; Jeewandara et al., 2015; Kamaladasa et al., 2016; Tissera et al., 2017; Inokuchi et al., 2018; Malavige et al., 2018). As mast cells reside in tissues and around blood vessels, it has been difficult to determine their infectivity rates by the DENV and phenotypic changes (St. John, 2013). As they also reside in the skin, they can be readily infected and activated during very early infection, when the mosquito injects the DENV into the dermis. Although certain mediators such as PAF and VEGF can also be produced by other cells types such as macrophages, monocytes and endothelial cells (Walterscheid et al., 2002), chymases and tryptases are only produced by mast cells (Krystel-Whittemore et al., 2015). Both chymase and tryptase have been shown to be high in patients with acute dengue during early illness and shown to be an important predictor of progression to severe dengue, suggesting that mast cells indeed do have an important role in disease pathogenesis (Tissera et al., 2017; Rathore et al., 2019). Chymase levels were shown to be higher in those who progressed to develop DHF after day 3 of illness (Rathore et al., 2020). However, it is not yet clear whether the differences between those with primary and secondary dengue associate with increased mast cell activation. Serum tryptase has been shown to be higher in patients with DHF and this rise was especially seen during day early illness (Jeewandara et al., 2017; Rathore et al., 2019). Tryptase has been shown to induce vascular leak by acting on endothelial gap junctions and was shown to induce plasma leakage and shock in animal models, suggesting that it indeed is likely to have an important role in inducing vascular leak in dengue (Rathore et al., 2019). In acute dengue, no differences were observed in tryptase levels in those with primary and secondary dengue (Jeewandara et al., 2017), suggesting that the role of DENV-specific antibodies activating mast cells through their FcγRs should be further investigated.

PAF levels were also shown to be high in those with vascular leak and are known to induce vascular leak (Jeewandara et al., 2015). A PAFR blocker (rupatadine), was shown to inhibit the reduction of trans endothelial electrical resistance and reduce the effect of sera of patients with DHF on endothelial tight junction (Malavige et al., 2018). In addition, it also showed dose dependent effects on reducing the rise in hematocrit in mouse models and reduction in the extent of plasma leakage in patients with acute dengue (Malavige et al., 2018). PAF is also known to activate NFκB and thereby induce production of many inflammatory cytokines and further contributing to disease pathogenesis (Choi et al., 2003). Although the source of PAF is not known in patients with acute dengue, it is possible that many different cell types including mast cells could be contributing to its production. PAF has varying effects on many other immune cells such as reduction in dendritic cell maturation, T cell anergy and reduction in T cell and depending on the levels either increase or a decrease in T cell proliferation (Kelesidis et al., 2015). Therefore, PAF appears to have many potential impacts on antiviral immunity other than inducing vascular leak; and therapeutics that block PAF are may be beneficial by acting through many pathways.

## Metabolic Disease and Innate Immunity

Individuals with comorbidities such as metabolic diseases and asthma are more likely to develop DHF and organ dysfunction (Guo et al., 2017; Pang et al., 2017; Wang et al., 2019). The presence of diabetes mellitus along with other metabolic diseases or poorly controlled diabetes mellitus were independent risk factors for development of severe dengue and shock (Lee et al., 2018). However, the mechanisms of severe dengue of those with comorbidities are not known. Patients with metabolic diseases have been shown to have low grade endotoxemia (Neves et al., 2013) due to gut microbial dysbiosis (Shin et al., 2015). Those with DHF have shown to have higher levels of serum lipopolysaccharide (LPS), which can result in activation of many innate immune cells through TLR-4 (van de Weg et al., 2012, 2013). While LPS alone induces many immune cells to produce inflammatory cytokines, LPS was shown to act synergistically with the DENV to further increase cytokine production and PAF, which can contribute to vascular leak and DHF (Kamaladasa et al., 2016). Therefore, such low grade endotoxemia in patients with metabolic disease, could be instrumental in inducing an unfavorable immune response to the DENV resulting in severe dengue. Gut microbial dysbiosis has shown to significantly influence the antiviral responses to vaccines (Lynn and Pulendran, 2018; Lynn et al., 2018). For instance, those who were given antibiotics just before the influenza vaccine, had reduced neutralizing and influenza specific IgG, and dysregulated immune responses, which was attributed to gut microbial dysbiosis (Hagan et al., 2019). Therefore, gut microbial dysbiosis may have a potential to induce severe dengue due to development of a poor quality, suboptimal antibody response.

Metabolic disease and obesity is associated with chronic, low grade inflammation and is associated with differences in monocyte subsets (Friedrich et al., 2019; Figueroa-Vega et al., 2020). While all CD14^+^CD16^+^(classical monocytes), CD14^dim^CD16^+^ (intermediate) and CD14^−^CD16^++^(non-classical) monocytes are susceptible to infection by the DENV, the non-classical monocytes are the main produced of inflammatory cytokines and IL-10, which are implicated in SD (Wong et al., 2012). *IFITI* and CD163 expression in CD14+CD16+ monocytes, was shown to precede the development of severe dengue (Zanini et al., 2018). As those with metabolic disease and obesity have a higher frequency of the non-classical types of monocytes (Friedrich et al., 2019), increased infection of this subtype by the DENV in such individuals could lead to severe dengue. Increase in mast cell numbers are also seen in adipose tissues of obese individuals, which are thought to contribute to low grade inflammation associated with metabolic syndrome and obesity (Elieh Ali Komi et al., 2020). Therefore, increase infection and activation of mast cells in those with metabolic disease could also lead to more severe disease.

## Effect of Innate Immune Responses on Virus Specific T Cells and B Cells

Although the role of T cells in dengue have been debated, emerging evidence suggests that they are likely to have a protective role (Weiskopf et al., 2013, 2015; Wijeratne et al., 2018, 2019). Highly cross reactive T cells are seen in acute dengue, which were thought to contribute to disease pathogenesis by production of high levels of inflammatory cytokines (Mongkolsapaya et al., 2003; Appanna et al., 2007; Dong et al., 2007). Indeed, it has been shown that higher levels of cross-reactive T cells that produce inflammatory cytokines were seen in those with more severe forms of illness rather than those with milder illness (Mongkolsapaya et al., 2006). However, such cross reactive T cells producing high levels of inflammatory cytokines were only seen 14 days since the onset of illness, when the patient was in the recovery phase (Mongkolsapaya et al., 2006). In fact, T cell responses were very low or absent in patients during the critical phase, where vascular leak and the cytokine storm occurs (Mongkolsapaya et al., 2006; Malavige et al., 2013b). This paucity of the DENV-specific T cells in acute dengue, could be due to the massive T cell apoptosis that has been reported in many studies (Mongkolsapaya et al., 2003; Malavige et al., 2012). Indeed, prolonged viremia and persistence of NS1 antigen is seen in those with severe dengue (Wang et al., 2006; Guilarde et al., 2008; Adikari et al., 2016), while early appearance of DENV-specific T cells was associated with milder disease and early clearance of the virus (Wijeratne et al., 2018). Patients with DHF had significantly less DENV-specific T cell responses (Wijeratne et al., 2018), than those with DF and the virus specific T cell responses were restored *in vitro*, with IL-10 blockade (Malavige et al., 2013b). Many studies have shown that T cell responses are absent or suboptimal in those with DHF during acute illness (Mongkolsapaya et al., 2006; Chau et al., 2008). Since early appearance of IL-10 was the main cytokine that was associated with subsequent progression to severe dengue, IL-10 is likely to also contribute to disease pathogenesis by inhibiting virus specific T cell responses and therefore, a delay in virus clearance.

The expansion of non-classical CD14^+^CD16^+^ monocytes has shown to associate with severe dengue (Kwissa et al., 2014; Zanini et al., 2018). DENV-infected CD14^+^CD16^+^ monocytes were found to be more efficient at stimulating resting B cells to transformation into plasmablasts by production of BAFF and APRIL (Kwissa et al., 2014). Increase in the frequency of plasmablasts have shown to associate with dengue disease severity (Wrammert et al., 2012; Appanna et al., 2016; Wijesinghe et al., 2020). Although the mechanisms by which the plasmablasts contribute to disease pathogenesis is unclear, it is possible that the increased antibody production by these cells, could contribute to severe disease by ADE.

## Summary

There is increasing evidence that a dysfunctional innate immune response leads to severe dengue by impaired production of interferons and increased production of inflammatory cytokines and lipid mediators. This dysregulated and aberrant immune response leads to reduced clearance of the virus, and severe dengue by inducing a vascular leak and by inducing excessive inflammation. Individuals with comorbid illnesses could be prone to more severe dengue due to low grade endotoxemia, gut microbial dysbiosis, and an altered phenotype of innate immune cells. The immunosuppressive and inflammatory lipid mediators and altered phenotype of monocytes are likely to further act on T cells and B cells leading to an impaired adaptive immune response to the virus. Therefore, in order to identify therapeutic targets for treatment of dengue, it would be important to further characterize these mechanisms in order for early intervention.

## Author Contributions

GM and GO: conceptualization, critical review, and writing the manuscript. CJ: writing the manuscript. All authors: contributed to the article and approved the submitted version.

## Conflict of Interest

The authors declare that the research was conducted in the absence of any commercial or financial relationships that could be construed as a potential conflict of interest.
